# Comparison of Antioxidant and Anticancer Properties of Soft Coral-Derived Sinularin and Dihydrosinularin

**DOI:** 10.3390/molecules26133853

**Published:** 2021-06-24

**Authors:** Sheng-Chieh Wang, Ruei-Nian Li, Li-Ching Lin, Jen-Yang Tang, Jui-Hsin Su, Jyh-Horng Sheu, Hsueh-Wei Chang

**Affiliations:** 1 Department of Biomedical Science and Environmental Biology, Ph.D. Program in Life Sciences, College of Life Sciences, Kaohsiung Medical University, Kaohsiung 80708, Taiwan; u107851101@gap.kmu.edu.tw (S.-C.W.); runili@kmu.edu.tw (R.-N.L.); 2 Chi-Mei Foundation Medical Center, Department of Radiation Oncology, Tainan 71004, Taiwan; 8508a6@mail.chimei.org.tw; 3 School of Medicine, College of Medicine, Taipei Medical University, Taipei 11031, Taiwan; 4 Chung Hwa University of Medical Technology, Tainan 71703, Taiwan; 5 School of Post-Baccalaureate Medicine, Kaohsiung Medical University, Kaohsiung 80708, Taiwan; reyata@kmu.edu.tw; 6 Department of Radiation Oncology, Kaohsiung Medical University Hospital, Kaoshiung Medical University, Kaohsiung 80708, Taiwan; 7 National Museum of Marine Biology & Aquarium, Pingtung 944, Taiwan; x2219@nmmba.gov.tw; 8 Institute of Marine Biotechnology, National Dong Hwa University, Pingtung 90078, Taiwan; 9 Department of Marine Biotechnology and Resources, National Sun Yat-sen University, Kaohsiung 80424, Taiwan; 10 Doctoral Degree Program in Marine Biotechnology, National Sun Yat-sen University, Kaohsiung 80424, Taiwan; 11 Department of Medical Research, China Medical University Hospital, China Medical University, Taichung 40402, Taiwan; 12 Frontier Center for Ocean Science and Technology, National Sun Yat-sen University, Kaohsiung 80424, Taiwan; 13 Center for Cancer Research, Kaohsiung Medical University, Kaohsiung 80708, Taiwan; 14 Cancer Center, Kaohsiung Medical University Hospital, Kaohsiung Medical University, Kaohsiung 80708, Taiwan; 15 Institute of Medical Science and Technology, National Sun Yat-sen University, Kaohsiung 80424, Taiwan; 16 Department of Medical Research, Kaohsiung Medical University Hospital, Kaohsiung Medical University, Kaohsiung 80708, Taiwan

**Keywords:** antioxidant, cytotoxicity, soft coral, marine natural products, sinularin, dihydrosinularin

## Abstract

Marine natural products are abundant resources for antioxidants, but the antioxidant property of the soft corals-derived sinularin and dihydrosinularin were unknown. This study aimed to assess antioxidant potential and antiproliferation effects of above compounds on cancer cells, and to investigate the possible relationships between them. Results show that sinularin and dihydrosinularin promptly reacted with 2,2-diphenyl-1-picrylhydrazyl (DPPH), 2,2-azinobis (3-ethyl-benzothiazoline-6-sulfonic acid) (ABTS), and hydroxyl (^•^OH), demonstrating a general radical scavenger activity. Sinularin and dihydrosinularin also show an induction for Fe^+3^-reduction and Fe^+2^-chelating capacity which both strengthen their antioxidant activities. Importantly, sinularin shows higher antioxidant properties than dihydrosinularin. Moreover, 24 h ATP assays show that sinularin leads to higher antiproliferation of breast, lung, and liver cancer cells than dihydrosinularin. Therefore, the differential antioxidant properties of sinularin and dihydrosinularin may contribute to their differential anti-proliferation of different cancer cells.

## 1. Introduction

Oxidative stress affects cellular function. Changes in the oxidative status may generate peroxidation impacts on lipids, proteins, and RNA and regulate cell response, signaling, and metabolism [1,2]. Peroxidation of lipids, proteins, DNA, and RNA may damage their biological functions and provide mitochondrial dysfunction and apoptosis [3]. Peroxidations were reported to be associated with several diseases such as neurodegenerative [4], atherosclerosis [5], and kidney disorders [6]. Therefore, antioxidation would assist in avoiding such cellular and tissue damages [7,8].

Marine natural compounds are rich exogenous resources of antioxidants [9,10,11,12]. Exogenous antioxidants commonly show a bi-phase oxidative stress-modulating ability towards cancer cells [13]. Compounds with antioxidant ability show different mediations of cellular oxidative stress. Exogenous antioxidants provide oxidative stress-suppressing ability at physiological concentrations, however, show oxidative stress-promoting power at cytotoxic concentrations.

Soft corals contain many bioactive natural compounds [14] that show anticancer effects [15,16,17,18,19,20,21]. Sinularin was isolated from the soft corals *Sinularia flexibilis* [22] and *S. manaarensis* [23]. Similarly, dihydrosinularin was firstly isolated from the soft coral *S. flexibilis* [24]. The IUPAC names for sinularin and dihydrosinularin are (9*E*)-13-hydroxy-4,9,13-trimethyl-17-methylidene-5,15-dioxatricyclo[1 2.3.1.0^4,6^]octadec-9-en-16-one and (9*E*)-13-hydroxy-4,9,13,17-tetramethyl-5,15-dioxatricyclo[1 2.3.1.0^4,6^]octadec-9-en-16-one, respectively [25]. The main chemical difference between them is that sinularin possesses a conjugated double bond which lacks in dihydrosinularin.

Although sinularin and dihydrosinularin are similar marine natural compounds, they show different bioactivities if investigated as yet. The antiproliferation ability of sinularin was reported in several types of cancer cells [16,20,22,26,27,28,29]. However, the antiproliferation reports of dihydrosinularin are rare. Cytotoxicity of dihydrosinularin was reported with respect to lymphocytic leukemia [22], lung and colon cancer cells [30]. The antioxidant properties of these related compounds were rarely investigated. Most of those studies focused on bioactive compound identification and cancer cell cytotoxicity. They rarely reported the detailed anticancer mechanisms, especially the role of antioxidant properties providing anticancer effect.

The present study aims at the antioxidant properties through radical-scavenging activities and examines the antiproliferation effect to breast, lung, and liver cancer cells applying an ATP assay for the similar compounds sinularin and dihydrosinularin.

## 2. Results

### 2.1. Radical Scavenging Activity of 2,2-Diphenyl-1-picrylhydrazyl (DPPH)

**Figure 1:**
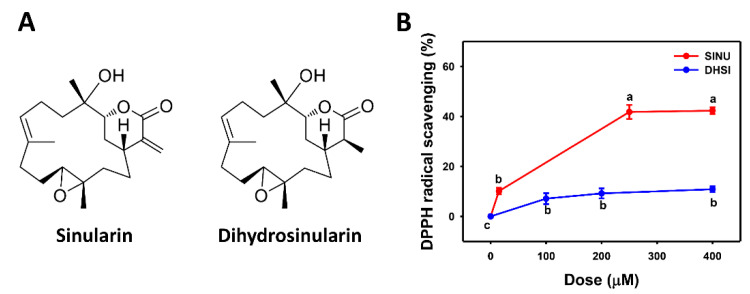
Structures and DPPH scavenging activities of sinularin and dihydrosinularin. (**A**) Structures. (**B**) DPPH scavenging activities. Data, mean ± SD (*n* = 3). DPPH scavenging activities for different concentrations of sinularin (SINU) and dihydrosinularin (DHSI) are compared to blank. According to multiple comparisons, data of the same characters showing non-overlap indicate significant differences (*p* < 0.05–0.001).

DPPH [31] is a common method for detecting in vitro antioxidant properties. Figure 1A shows the structures of sinularin and dihydrosinularin. In Figure 1B, the DPPH scavenging activity of sinularin increases below a threshold of 250 μM and reaches a plateau of 40% scavenging activity at 400 μM. DPPH scavenging activity of dihydrosinularin increases below 200 μM and reaches a plateau of 10% activity at 400 μM. Therefore, sinularin shows higher DPPH scavenging activity than dihydrosinularin.

### 2.2. Radical Scavenging Activity for 2,2-Azinobis (3-Ethyl-benzothiazoline-6-sulfonic Acid) (ABTS)

**Figure 2:**
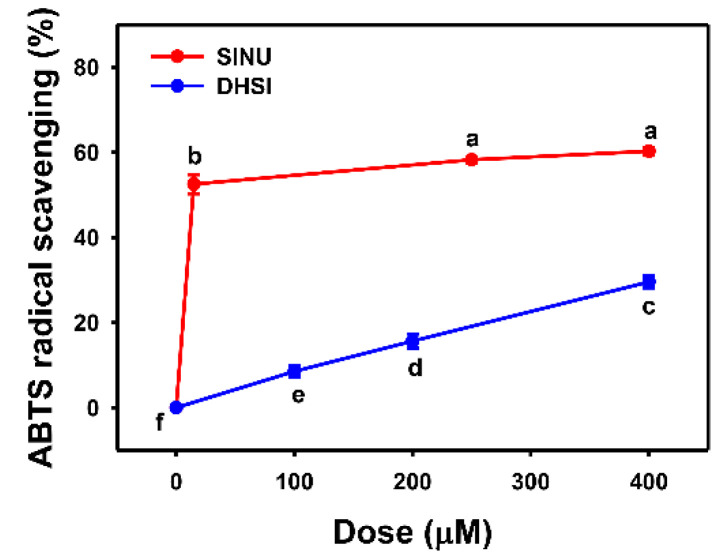
ABTS scavenging activities of sinularin and dihydrosinularin. Data, mean ± SD (*n* = 3). DPPH scavenging activities for different concentrations of sinularin (SINU) and dihydrosinularin (DHSI) are compared to blank. According to multiple comparisons, data of the same characters showing non-overlap indicate significant differences (*p* < 0.05–0.001).

ABTS^•+^ [32] is another in vitro antioxidant detection method. In Figure 2, the ABTS scavenging activity of sinularin dramatically increases to 50% at 15 μM and reaches a plateau of 60% activity above 250 μM. ABTS scavenging activities of dihydrosinularin increase in a dose-response manner within 400 μM, but it gets 30% at 400 μM. Therefore, sinularin shows higher ABTS scavenging activity than dihydrosinularin.

### 2.3. Hydroxyl (^•^OH) Radical Scavenging Activity

**Figure 3:**
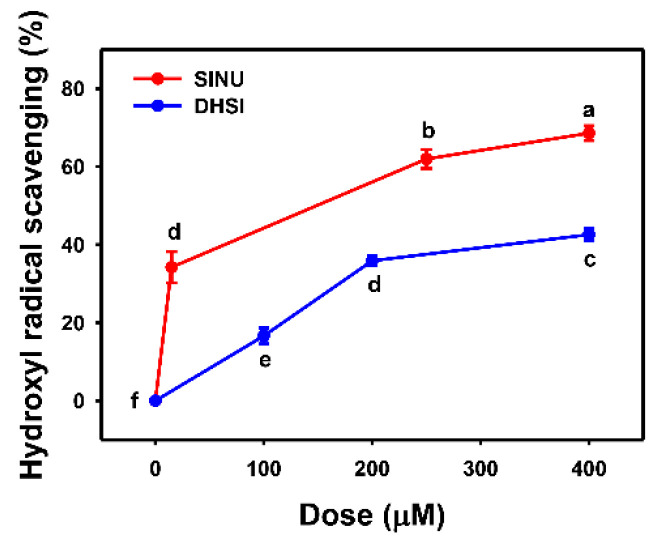
Hydroxyl scavenging activities of sinularin and dihydrosinularin. Data, mean ± SD (*n* = 3). DPPH scavenging activities for different concentrations of sinularin (SINU) and dihydrosinularin (DHSI) are compared to blank. According to multiple comparisons, data of the same characters showing non-overlap indicate significant differences (*p* < 0.05–0.001).

^•^OH initiates an early stage of lipid hydroperoxidation for producing the lipid radical to trigger lipid peroxidation [33]. Accordingly, measurement of ^•^OH radical scavenging activity was also used to assess in vitro antioxidant properties [34]. In Figure 3, the ^•^OH scavenging activity of sinularin dramatically increases to 35% at 15 μM and reaches plateaus of 60% and 70% activity at 250 and 400 μM, respectively. ^•^OH scavenging activities of dihydrosinularin increase in a dose-response manner within the 400 μM range, but it reaches only 40% at 400 μM. Therefore, sinularin shows higher ^•^OH scavenging activity than dihydrosinularin.

### 2.4. Ferric Ion (Fe^+3^)-Reducing Power

**Figure 4:**
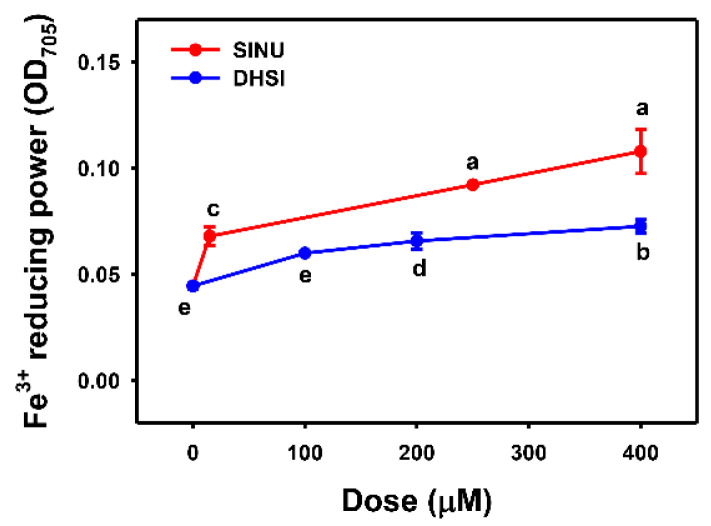
Ferric ions-reducing powers of sinularin and dihydrosinularin. Data, mean ± SD (*n* = 3). Ferric ions-reducing powers for different concentrations of sinularin (SINU) and dihydrosinularin (DHSI) are compared to blank. According to multiple comparisons, data of the same characters showing non-overlap indicate significant differences (*p* < 0.05–0.001).

Fe^+3^-reducing power is an iron-based in vitro antioxidant measurement [35]. In Figure 4, sinularin and dihydrosinularin increase Fe^+3^-reducing power in a dose-response manner. Sinularin shows higher Fe^+3^-reducing powers than dihydrosinularin.

### 2.5. Ferrous Ion (Fe^+2^)-Chelating Capacity

**Figure 5:**
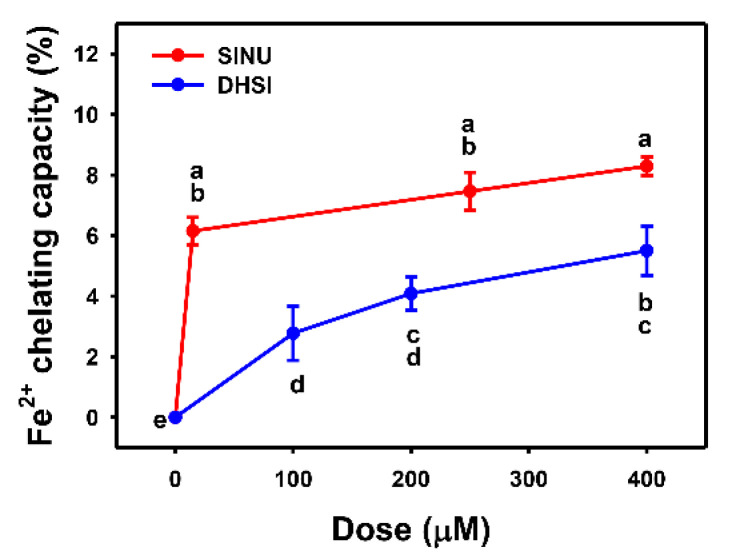
Ferrous ions-chelating capacities of sinularin and dihydrosinularin. Data, mean ± SD (*n* = 3). Ferrous ions-chelating capacities for different concentrations of sinularin (SINU) and dihydrosinularin (DHSI) are compared to blank. According to multiple comparisons, data of the same characters showing non-overlap indicate significant differences (*p* < 0.05–0.001).

Fe^+2^-chelating capacity is also the iron-based in vitro antioxidant detection [35]. In Figure 5, the Fe^+2^-chelating capacity of sinularin dramatically increases to 6% at 15 μM and reaches a plateau for 7% activity larger than 250 μM. Dihydrosinularin increases Fe^+2^-chelating capacity in a dose-response manner within 400 μM, but it comes 4% at 400 μM. Therefore, sinularin shows a higher Fe^+2^-chelating capacity than dihydrosinularin.

### 2.6. Cell Viabilities of Several Drug-Treated Cancer Cell Lines

We applied an ATP assay as a mitochondrial function-based detection of cell viability [36]. In triple-negative breast MDA-MB-231, lung H1299 cells, and liver HA22T/VGH cancer cells, the IC_50_ values of sinularin at 24 h ATP assays were 32, 2, and 12 μM, respectively. In comparison, the IC_50_ values of dihydrosinularin in MDA-MB-231, H1299, and HA22T/VGH cells were 60, 70, and 120 μΜ, respectively. Therefore, sinularin shows higher antiproliferation ability than dihydrosinularin to breast, lung, and liver cancer cells.

## 3. Discussion

The main difference between sinularin and dihydrosinularin is that sinularin, but not dihydrosinularin, has a conjugated double bond within the carbonyl group of the lactone ring. However, the antioxidant and antiproliferation abilities of sinularin and dihydrosinularin were rarely investigated. Differences between sinularin and dihydrosinularin were evaluated in the present study. The possible reasons why sinularin and dihydrosinularin exhibit differential antioxidant and antiproliferation abilities were discussed.

Different double bonds may contribute to the differential antioxidant effect. For example, polyunsaturated fatty acids (PUFA) containing many double bonds and bis-allylic hydrogen atoms are easily oxidized and show antioxidant abilities [37]. Double bonds at different positions may have differential antioxidant powers. For example, *iso*-moracin C contains the double bond at conjugation position, which is not the case in moracin C. *Iso*-moracin C shows higher antioxidative activity than moracin C [38].

Free radicals tend to directly attack the conjugated double bonds [39], indicating their potential antioxidant properties. For example, conjugated double bonds of terpenes contribute to the high antioxidant properties of free radical scavenging [40]. In contrast, blocking conjugated double bonds suppresses the antioxidant power of monoterpenes [41]. Accordingly, chemicals with conjugated double bonds exhibit a relative higher redox-related antioxidant power than chemicals without conjugated double bonds. However, above studies focused on the relationship of structure and antioxidant effects without aiming at possible anticancer effects.

The current study reports for the first time the antioxidant abilities of the soft-coral-derived compounds sinularin and dihydrosinularin using several well-established assays. Although both sinularin and dihydrosinularin showed antioxidant properties, sinularin exhibits higher DPPH, ABTS^•^^+^, and ^•^OH scavenging activity as well as Fe^+3^-reducing power and Fe^+2^-chelating capacity than dihydrosinularin. We conclude that the high antioxidant ability of sinularin is probably caused from its conjugated double bond, which becomes a single bond with dihydrogen atoms in dihydrosinularin (Figure 1A).

Several marine natural products with antioxidant abilities show anticancer effects. This finding is confusing because the antioxidant is regarded as a protector from several cellular oxidative stress damages. Recently, a dual role of antioxidants has been proposed. At low concentrations of antioxidants, oxidative stress for cancer cell proliferation can be reduced. In contrast, lethal concentrations of antioxidants may induce oxidative stress that causes antiproliferation of cancer cells. Similarly, a famous natural antioxidant grape seed extract shows antioral cancer effect with oxidative stress generation at high concentrations but not at low concentrations [42]. Therefore, the exogenous antioxidant exhibits a concentration-dependent modulation of cellular oxidative stress.

**Figure 6:**
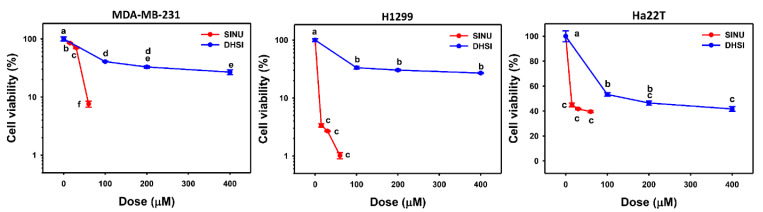
Cell viability of triple-negative breast and liver cancer cells following sinularin and dihydrosinularin treatments. Breast MDA-MB-231, lung H1299, and liver Ha22T cancer cells were treated with sinularin (SINU) and dihydrosinularin (DHSI) for 24 h incubation. Subsequently, their viabilities were determined by ATP assay (data, mean ± SD (*n* = 3)). According to multiple comparisons, data of the same characters showing non-overlap indicate significant differences (*p* < 0.05–0.001).

As described above, lethal concentrations of antioxidants have oxidative stress-generating potential. Since sinularin exhibits higher antioxidant effects than dihydrosinularin, it is expected that sinularin may have a higher potential to generate oxidative stress at lethal concentrations and leads to the death of cancer cells. This notion was supported by the finding that dihydrosinularin triggered oxidative stress that inhibited cell proliferation of oral [28] and renal [29] cancer cells. Consistently, the antiproliferation ability of sinularin is higher than dihydrosinularin in the example of breast and lung cancer cells (Figure 6). The higher antiproliferation ability of sinularin are explained here with its higher antioxidant ability than dihydrosinularin when high concentrations are applied to cancer cells.

In addition to their different antioxidant abilities, the more potent cytotoxicity of sinularin than dihydrosinularin is explained here with the ability of the conjugated exocyclic double bond of sinularin to form an adduct through a “Michael addition” with the thiol group of specific enzymes. This possible mechanism warrants more detailed investigations in the future.

## 4. Materials and Methods

### 4.1. Preparation of Two Bioactive Diterpenoids and Chemicals

Following a previous study [23], the lyophilized bodies of the soft coral *S. manaarensis* were minced and extracted with ethyl acetate, and the crude extract was chromatographed to yield both cembranoids sinularin and dihydrosinularin in high purity, as confirmed by NMR spectra of the two compounds. Both of them were dissolved in ethanol for antioxidant assays and in dimethyl sulfoxide (DMSO) for cell viability assays. All chemical reagents were purchased from Sigma-Aldrich Inc (St. Louis, MO, USA).

### 4.2. DPPH Radical Scavenging Activity

The scavenging effect for DPPH was assessed as mentioned [32,43]. An amount of 125 μM DPPH (dissolved in ethanol) was equally mixed and reacted with either sinularin or dihydrosinularin (dissolved in ethanol) in the darkness for 30 min. A multiplate reader (800 TS, BioTek Instruments, Inc., Winooski, VT, USA) measured the reaction at 517 nm.

### 4.3. ABTS^•+^ Radical Scavenging Activity

The scavenging effect for ABTS was assessed as mentioned [32,44]. 7.4 mM ABTS^•^^+^/2.6 mM persulfate solution was equally mixed and reacted with sinularin or dihydrosinularin (dissolved in ethanol) in the darkness for 15 min. A multiplate reader measured the reaction at 734 nm.

### 4.4. ^•^OH Radical Scavenging Activity

The scavenging effect for ^•^OH radical was assessed as mentioned [34]. An amount of 690 μL of 2.5 mM 2-deoxyribose (in phosphate buffer (0.2 M, pH 7.4) and 100 μL of 0.1 mM FeCl_3_ (dissolved in 1.04 mM EDTA) were mixed with 100 μL of sinularin or dihydrosinularin (dissolved in ethanol). Subsequently, they were allowed to react with the mixture (100 μL of 10 mM ascorbic acid and 10 μL of 0.1 M H_2_O_2_) at 37 °C for 10 min. Finally, they were combined with a mixture (500 μL of 1% thiobarbituric acid (TBA) and 1000 μL of 2.8% trichloroacetic acid (TCA)) for 10 min. A multiplate reader measured the reaction at 532 nm.

### 4.5. Fe^+3^-Reducing Power

Reducing power was assessed as mentioned [31,44]. 100 μL of 0.2 M phosphate buffer (pH 6.6) and 100 μL of 1% potassium ferricyanide were reacted with 100 μL of sinularin or dihydrosinularin (dissolved in ethanol) at 50 °C for 20 min. Subsequently, they were reacted with the mixture (500 μL of 2% TCA and 400 μL of 0.1% FeCl_3_) for 15 min. A multiplate reader measured the reaction at 705 nm.

### 4.6. Fe^+2^-Chelating Capacity

Chelating capacity was assessed as mentioned [44]. 740 μL of deionized water was mixed with 20 μL of 2 mM FeCl_2_ to react with 200 μL of sinularin or dihydrosinularin (dissolved in ethanol). Subsequently, they were reacted in shaking with 40 μL of 5 mM ferrozine for 10 min. A multiplate reader measured the reaction at 562 nm.

### 4.7. Cell Viability

Human triple-negative breast (MDA-MB-231) and lung (H1299) cancer cell lines were obtained from ATCC, and liver (HA22T/VGH) cancer cell lines were obtained from Bioresource Collection and Research Center (Hsinchu, Taiwan). These cell lines were maintained by DMEM medium (Gibco, Grand Island, NY, USA) containing 10% fetal bovine serum (FBS) and antibiotics as previously described [45]. Cell viabilities following sinularin and dihydrosinularin exposure were assessed at 24 h incubation using an ATP detection kit (PerkinElmer Life Sciences, Boston, MA, USA) [46]. A microplate luminometer (Berthold Technologies GmbH & Co., Bad Wildbad, Germany) measured the light reaction.

### 4.8. Statistical Analysis

The analysis of variance (ANOVA) followed by a HSD Post-Hoc Test were applied for significance analysis. Data showing non-overlapping the same characters indicate a significant difference in multiple comparisons.

## 5. Conclusions

Antioxidants exhibit a comprehensive biological function. A similar chemical structure may demonstrate different antioxidant power. For the first time, the current study reported the antioxidant ability of marine coral-derived sinularin and dihydrosinularin by using several well-established in vitro assays. The antioxidant properties of sinularin were higher than those of dihydrosinularin. This high antioxidant power of sinularin at lethal concentrations may generate high oxidative stress. This explains our observation that sinularin has higher antiproliferation abilities than dihydrosinularin in breast, lung, and liver cancer cells.

## Data Availability

Not applicable.

## Sample Availability

Samples of the compounds are not available from the authors.
